# Demographic, clinical, treatment, outcome, and comorbidities of patients with relapsing polychondritis: experience from Brazilian tertiary center, and literature review

**DOI:** 10.1186/s42358-025-00456-5

**Published:** 2025-05-14

**Authors:** Patricia Pilar Lury Ortale Ueda, Luiz Antonio Leandrini Komati, Samuel Katsuyuki Shinjo

**Affiliations:** 1 Division of Rheumatology, Faculdade de Medicina FMUSP, Universidade de Sao Paulo, Sao Paulo, SP Brazil; 2 Disciplina de Reumatologia, Faculdade de Medicina, Universidade de Sao Paulo, Av. Dr. Arnaldo, 455, 3° andar, sala 3184 - Cerqueira César, Sao Paulo, BR CEP: 01246-903 Brazil

**Keywords:** Systemic autoimmune disease, Relapsing polychondritis, Revision

## Abstract

**Background:**

Due to the rarity of relapsing polychondritis (RP), we described the demographic, clinical, treatment, outcomes, and comorbidities of patients with RP from our tertiary service. Additionally, a literature review was conducted.

**Methods:**

A total of 47 Brazilian patients with RP between 2000 and 2024 were analyzed. All patient data were collected from pre-parametrized and pre-standardized electronic medical records. A literature review using PubMed with “relapsing polychondritis” as the search term included 25 articles after applying the strict exclusion criteria.

**Results:**

A total of 47 patients were evaluated. The median age was 40 (34–51) years, with a female-to-male ratio of 1.4:1, and 89.4% were of white ethnicity. The median time from symptom onset to diagnosis was 39 months and the median follow-up duration was 7 years. Ear cartilage biopsy was performed in 12.8% of cases. The clinical manifestations included auricular chondritis, arthralgia, and ocular involvement. Approximately half of the patients had hypertension and dyslipidemia, one-third had diabetes mellitus, and one-fifth had hypothyroidism. Tracheostomy and cochlear implantation were required in 12.8% and 6.4% of the patients, respectively. Disease outcomes showed that 46.8% of patients were in remission, 29.8% had active disease, and 25.5% were controlled with immunosuppressive therapy. Mortality occurred in 6.4% of the cases. In the literature review, 25 studies were analyzed, most of which originated in Asia. Studies have reported the classical manifestations of RP, such as auricular chondritis, arthritis, and ocular involvement. The median age of the patients with RP was similar across studies, averaging 46.4 years, with a predominance of female patients. A comparison with the literature showed consistency in clinical manifestations, particularly auricular chondritis and septum nasal chondritis, although few studies have explored comorbidities, disease evolution, and outcomes.

**Conclusions:**

The reviewed articles described classical clinical manifestations, but few articles reported data on other manifestations and comorbidities that can occur in RP. Our study provided new insights by mapping symptom evaluations, thereby enhancing the understanding of disease evolution. Understanding and characterizing RP will allow for better assistance in its diagnosis and follow-up.

## Introduction

Relapsing polychondritis (RP) is a rare systemic autoimmune disease characterized by the inflammation of cartilaginous and/or proteoglycan-rich tissues [1, 2]. In addition to classic ear involvement, the disease can affect other organs and systems [1–3], such as the eyes, heart, joints, blood vessels, and the upper and lower airways [4].

The annual incidence of RP is 0.71 cases/million, affecting both sexes. Mortality is twice as high as that in the general population [5].

Regarding the evolution of RP, recurrent inflammation leads to tissue destruction [6] and results in neurological, ocular, dermatological, and other manifestations [7]. Clinical manifestations include inflammation of certain tissues or organs, such as chondritis (auricular, nasal, costal, joints, etc.), episcleritis, scleritis, uveitis, corneal ulcers, and retinal vasculitis [8].

Until now, there are few epidemiological studies with significant samples of RP patients [1, 4, 11–13, 22−42]. Furthermore, these studies were limited to patients from the Northern Hemisphere, especially Asia [11–13, 23, 25, 27, 33, 37, 39, 42], Europe [1, 4, 35, 38], and North America [26, 34, 36, 40, 43]. However, only one study has been conducted in the Southern Hemisphere, particularly in South America, with a small sample size [22].

Therefore, we aimed to describe the demographic and clinical features, evolution, and comorbidities of a representative sample of patients with RP undergoing follow-up at a Brazilian tertiary center. Second, a literature review was conducted.

## Patients and methods

This retrospective cohort study included patients with RP who underwent regular outpatient follow-up at a tertiary rheumatology service from 2000 to 2024.

Patients with RP were included according to the diagnostic criteria of McAdam et al. [10]

The following pre-parameterized and pre-standardized information was extracted from the patients’ medical records:

- Demographic and anthropometric data: Current age, sex, and ethnicity;
- Clinical: Age at disease onset and duration;
- Drug therapy: Use of remote and current medications (glucocorticosteroids, immunosuppressants, immunomodulators, and immunobiologicals);
- Clinical evolution and current status of the disease (remission, activity, or control with immunosuppressive therapy), basing on clinical and/or laboratory parameters;
- Comorbidities: presence of other autoimmune rheumatic diseases; systemic arterial hypertension (or use of antihypertensive medication), dyslipidemia, diabetes mellitus, hypothyroidism, chronic obstructive lung disease, heart failure, myocardial infarction, stroke, and neoplasm;
- Habits: Smoking (if present or absent);
- Mortality.

### Literature review

This search was based on the PubMed database, using “relapsing polychondritis” as the descriptor in the title or abstract. The analysis of the articles was independently performed by two authors. Duplicate studies were initially excluded, followed by case reports with fewer than 50 cases, literature review articles, conference summaries and annals, comments and errata of articles and book chapters, articles in the English language, and articles not available.

### Statistical analysis

The results were presented as median (interquartile range) or mean ± standard deviation for continuous variables, and as numbers (%) for categorical variables.

## Results

### Patients with RP in the present study

A total of 47 patients with RP were evaluated, with a median age at disease onset of 40 (34–51) years. The female-to-male ratio was 1.4:1, and white ethnicity was observed in 42 (89.4%) patients. The median duration between diagnosis and symptom onset was 39 (38–50) months, and the median follow-up duration was 7 (2–10) years. Ear cartilage biopsy was performed in six (12.8%) cases.

The initial and cumulative clinical manifestations of the 47 patients with RP are shown in Table 1, and the previous (cumulative) and current treatments are shown in Table 2.

**Table 1:** Initial and follow-up/cumulative clinical manifestations of 47 patients with relapsing polychondritis

Clinical manifestations	Initial	Follow-up/ Cumulative
Auricular chondritis	47 (100)	47 (100)
Arthralgia	15 (31.9)	25 (53.2)
Arthritis	13 (27.7)	16 (34.0)
Septum nasal chondrites	11 (23.4)	17 (36.2)
Nasal bridge	0	5 (10.6)
Hearing loss	10 (21.3)	13 (27.7)
Uveitis	9 (19.1)	14 (29.8)
Episcleritis	7 (14.9)	8 (17.0)
Laryngeal disorder	5 (10.6)	5 (10.6)
Tracheal disorder	2 (4.3)	10 (21.3)
Bronchitis	3 (6.4)	6 (12.8)
Costochondritis	7 (14.9)	10 (21.3)
Subglottic stenosis	3 (6.4)	7 (14.9)
Vestibular disorder	2 (4.3)	8 (17.0)
Neurological disorder	2 (4.3)	5 (10.6)
Cardiac disorder	0	1 (2.1)
Renal disorder	0	1 (2.1)

Results expressed as frequency (%)

**Table 2:** Previous (cumulative) and current treatment of patients with 47 relapsing polychondritis

Treatment	Previous (cumulative)	Current
Glucocorticoid		
Pulse therapy with MP	11 (23.4)	0
Using	39 (83.0)	17 (36.2)
Dose (mg/day)	-	15 (15–30)
Intravenous immunoglobulin	4 (8.5)	0
Cyclophosphamide	14 (29.8)	0
Methotrexate	39 (83.0)	11 (23.4)
Azathioprine	21 (44.7)	8 (17.0)
Mycophenolate mofetil	10 (21.3)	1 (2.1)
Antimalarial	7 (14.9)	1 (2.1)
Leflunomide	6 (12.8)	1 (2.1)
Dapsone	3 (6.4)	0
Cyclosporine	4 (4.5)	1 (2.1)
Thalidomide	2 (4.3)	1 (2.1)
Immunobiological	12 (25.5)	7 (14.9)

Data are expressed as median (interquartile 25th – 75th) or frequency (%)

MP: methylprednisolone

The cumulative clinical manifestations of the 47 patients with RP are described in Table 3 together with those described in the literature.

**Table 3:** General features of study with relapsing polychondritis

Authors Ref	Year	Study	Cases (*n*)	Age at RP diagnosis (years)	F: M ratio	Auricular chondritis (%)	Arthralgia (%)	Arthritis (%)	Septum nasal chondritis (%)	Nasal bridge (%)	Hearing loss (%)	Ocular involvement (%)	Uveitis (%)	Episcleritis (%)	Laryngeal involvement (%)	Tracheobronchial (%)	Laryngotracheitis (%)	Costochondritis (%)	Subglottic stenosis (%)	Vestibular involvement (%)	Neurological involvement (%)	Cardiac involvement (%)	Renal involvement (%)
Current study	2024	Brazil	47	40	1.4:1	87.2	53.2	34	36.2	10.6	27.7	46.8	29.8	17	10.6	34.0	31.9	21.3	14.9	10.6	10.6	2.1	2.1
Zeuner et al. [20]	1997	Deutschland	62	46.6	0.7:1	93.5	-	-	56.5	-	-	49	-	-	-	-	-	2.3	-	-	9.7	-	7.9
Ernst et al. [8]	2009	USA	145	-	-	-	-	-	35.2	-	-	32.5	-	-	-	-	-	-	5.5	-	-	-	-
Shimizu et al. [2]	2015	Japan	239	52.7	0.9:1	78.2	-	-	-	39.3	61.7	46.1	-	-	-	-	-	-	-	-	9.6	7.1	9.2
Shimizu et al. [15]	2015	Japan	239	52.7	0.9:1	78	-	-	15.3	-	-	-	-	-	-	-	-	19.4	-	-	-	-	-
Lin et al. [4]	2016	China	158	45.3	0.7:1	68	-	56	-	-	25	44	-	-	-	-	69	15	-	-	-	-	3
Horváth et al. [5]	2016	Hungary	233	-	1:1	-	-	-	-	-	-	57.3	-	-	-	-	-	-	-	-	-	-	-
Dion et al. [17]	2016	France	142	-	1.7:1	89	66.2	33.1	-	-	27	56	13	36	43	22	-	40	-	-	-	-	-
Ferrada et al. [18]	2018	USA	304	43,2	6.4:1	86	-	-	63	-	34	53	-	-	-	74	-	-	15	-	-	-	-
Shimizu et al. [24]	2018	Japan	239	-	0.9:1	-	-	-	39	-	-	-	-	-	-	-	-	-	-	-	9.6	-	6.7
Zhang et al. [19]	2019	China	87	45.9	1.7:1	57.5	-	14.9	16.1	-	9.2	50	-	-	-	-	42.5	47	-	-	1.1	-	6.5
Shimizu et al. [21]	2020	Japan	229	-	0.9:1	-	-	-	40	-	-	-	-	-	-	-	-	-	-	-	12	-	-
Chen et al. [3]	2021	China	295	41	1:1	-	40	-	-	-	-	-	-	-	-	-	-	-	-	-	12	-	-
Zhang et al. [9]	2021	China	126	47.1	0.9:1	59.6	-	12.7	-	-	9.5	47	-	-	13.5	38.1	-	2.4	-	-	-	-	-
Cao et al. [12]	2021	China	181	-	0.8: 1	83.2	-	-	61.9	-	-	34.8	-	-	-	-	77.3	58	-	-	3.2	-	-
Ferrada et al. [16]	2021	USA	73	43	5.6:1	14	-	-	39	-	26	-	-	-	-	-	-	-	-	-	12	-	-
Ferrada et al. [26]	2021	USA	85	-	5.5:1	-	-	91	84	-	29	-	-	-	-	-	-	85	-	-	-	-	-
Nakajima et al. [10]	2022	-	117	43.2	2.3:1	78.6	-	-	47.9	-	-	19.8	-	-	-	59.9	-	-	-	-	4.8	-	-
Shimizu et al. [11]	2022	Japan	190	-	0.9:1	-	-	-	26.3	-	-	43.2	-	-	-	-	-	-	-	-	45	-	7.9
Wang et al. [13]	2022	China	72	55.2	0.7:1	62.4	-	8.3	-	-	-	-	-	-	11.1	-	-	-	-	-	-	-	-
Sangle et al. [6]	2023	Great Britain	68	48	2.1:1	58.8	-	20	-	15	20.6	-	-	-	-	-	73.5	-	5.9	-	-	-	-
Handa et al. [7]	2023	Japan	77	-	1.2:1	22.1	-	60.3	64.7	-	-	-	-	-	-	-	-	-	-	-	-	-	-
Luo et al. [22]	2023	USA	66	38	3.3:1	64	-	-	76	-	-	24	-	-	-	-	-	-	-	-	-	-	-
Yi et al. [23]	2023	China	52	48	0.6:1	-	-	-	-	-	-	-	-	-	-	-	-	42	-	-	-	-	-
Yin et al. [25]	2023	China	187	-	1:1	-	48.4	-	56.3	-	-	58.4	-	-	47.9	79.2	-	-	-	-	14.2	-	2.5
Yin et al. [27]	2023	China	146	-	-	21.9	41.8	-	-	-	16.8	18.9	-	-	-	-	-	-	-	-	4.9	-	1.2

Regarding surgery, six (12.8%) and three (6.4%) patients in the present study underwent tracheostomy and cochlear implantation, respectively (Table 4).

**Table 4:** Surgery, comorbidities, habits, and outcome of patients with relapsing polychondritis

Authors (Ref)	Tracheostomy (%)	Cochlear implant (%)	Arterial hypertension (%)	Dyslipidaemia (%)	Diabetes mellitus (%)	Hypothyroidism (%)	Myelodisyplasta syndrome (%)	Chronic obstructive lung disease (%)	Heart failure (%)	Myocardial infartion (%)	Stroke (%)	Smokers (%)	Neoplasia (%)	Mortality (%)
Current study	12.8	6.4	48.9	46.8	31.9	19.1	4.3	4.3	2.1	0	0	21.3	0	4.3
Zeuner et al. [20]	-	-	-	-	-	-	-	-	-	-	-	-	-	
Ernst et al. [8]	2	-	-	-	-	-	-	-	-	-	-	-	-	-
Shimizu et al. [2]	17.6	-	-	-	-	-	-	-	-	6.5	-	-	-	-
Shimizu et al. [15]	-	-	-	-	-	-	2.1	-	-	1.2	2.1	-	-	-
Lin et al. [4]	-	-	-	-	-	-	-	-	-	0.6	-	-	-	6
Horváth et al. [5]	-	-	-	-	16	-	-	-	-	-	-	-	27	-
Dion et al. [17]	-	-	-	-	-	-	-	-	-	-	-	-	-	11
Ferrada et al. [18]	-	-	-	-	-	-	-	-	-	-	-	-	-	-
Shimizu et al. [24]	-	-	-	-	-	-	2.1	-	-	-	-	-	-	-
Zhang et al. [19]	-	-	-	-	-	-	1.1	-	-	-	-	-	-	
Shimizu et al. [21]	-	-	-	-	-	-	-	-	-	-	-	-	-	-
Chen et al. [3]	14.9	-	-	-	-	-	-	-	-	-	-	-	2	8.8
Zhang et al. [9]	-	-	-	-	-	-	0.8	-	-	-	-	-	-	14.3
Cao et al. [12]	-	-	13.2	-	15.5	-	-	-	-	-	-	42.5	-	-
Ferrada et al. [16]	1	-	-	-	-	-	-	-	-	-	-	-	-	-
Ferrada et al. [26]	-	-	-	-	-	-	-	-	-	-	-	-	-	4
Nakajima et al. [10]	-	-	-	-	-	-	-	-	-	-	-	-	-	-
Shimizu et al. [11]	2.6	-	-	-	-	-	-	-	-	-	-	-	-	1.6
Wang et al. [13]	-	-	-	-	-	-	-	-	-	5.6	-	40.3	-	6.9
Sangle et al. [6]	-	-	-	-	19	-	-	-	-	-	-	-	-	18
Handa et al. [7]	32.4	-	-	-	-	-	-	-	-	-	-	-	-	22
Luo et al. [22]	-	-	-	-	-	-	-	-	-	-	-	-	-	-
Yi et al. [23]	-	-	-	-	-	-	-	-	-	-	-	-	-	-
Yin et al. [25]	-	-	8.4	-	3.7	-	-	-	-	-	-	39.3	-	-
Yin et al. [27]	9	-	7.3	-	13	-	-	-	-	-	-	-	-	-

One patient developed undifferentiated spondylarthritis. Approximately 50% of the patients exhibited systemic arterial hypertension and dyslipidemia were present in approximately half of our patients, whereas diabetes mellitus and hypothyroidism were observed in one-third and one-fifth of the patients, respectively (Table 4). Two (4.3%) patients had chronic obstructive lung disease, one (2.1%) patient had heart failure, one (2.1%) patient had myocardial infarction, and no patients had stroke. Five (10.6%) patients had history of neoplasm (two cases of myelodysplasia syndrome; one case of prostate radiotherapy, melanoma resection, and adenocarcinoma colon resection).

Smoking was reported in 10 (21.3%) patients.

RP mortality in our sample occurred in three (6.4%) cases.

Regarding the disease status of our patients at the last consultation, 22 (46.8%) patients presented with disease in remission, 14 (29.8%) patients had disease activity, and 12 (25.5%) patients had disease controlled with immunosuppressive therapy.

### Literature review

**Fig. 1:**
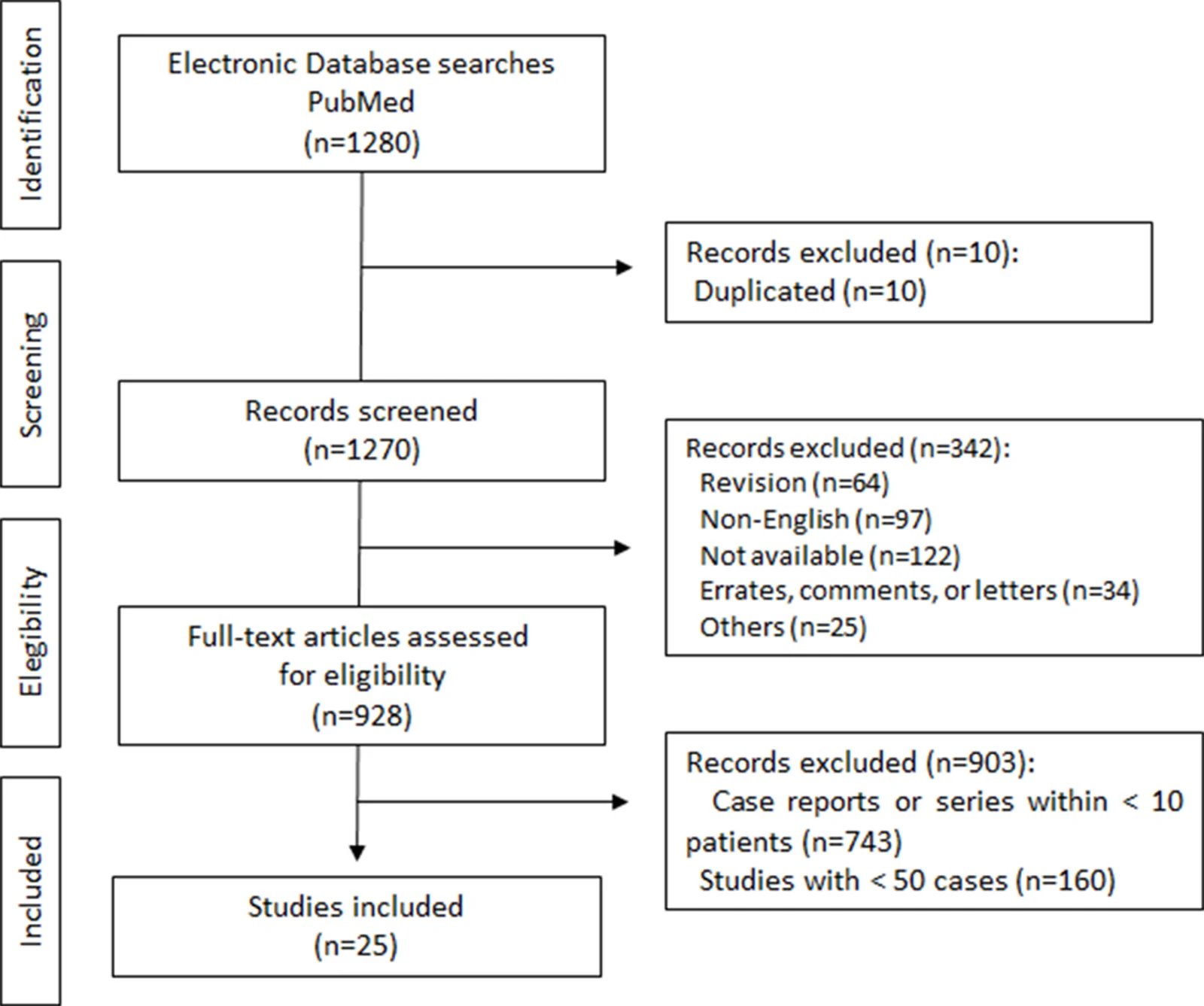
Flowchart of the present study

A total of 25 articles published between 1997 and 2024 were selected for the present study (Fig. 1; Tables 3 and 4). These studies are geographically diverse, but most are from Asia, mainly from China [11–13, 24, 27, 30, 31, 41], followed by Japan [23, 25, 28, 29, 33, 39, 42]. Other studies were conducted in the USA [26, 34, 36, 40, 43], Deutschland [38], France [35], and Great Britain [1]. In terms of sex distribution, nine studies primarily featured female participants [1, 25, 28, 34, 37, 40, 43], three had an equal representation of male and female participants [11, 13], and two specified the sex distribution [12, 26]. The remaining studies included a higher number of male participants [23, 24, 27, 29–31, 33, 38, 39, 41, 42]. This distribution highlights the predominance of studies from Asia, indicating the lack of a comprehensive global scope. The average age of the participants across the studies, based on the data provided by 14 articles [1, 13, 23, 24, 27, 28, 31, 33, 34, 36–38, 40, 41], was 46.4 years.

The reviewed articles reported clinical manifestations reported in the reviewed articles varied, with specific manifestations noted at both initial onset and follow-up stages.

Auricular chondritis has been observed in many studies [1, 3, 10–12, 20, 22–25, 27, 30, 33, 38], but few have been divided into initial [27] or follow-up [23, 24] onset. Arthralgia was mostly shown in the general [11–13, 35] and only one showed initial onset [13]. Arthritis was present in the general form [1, 24, 25, 27, 31, 35, 37, 43], but only two had an initial onset [24, 27] and one had a follow-up onset [24]. Septum nasal chondritis has been reported in several studies [11, 25, 26, 28–30, 33, 34, 36–40, 42, 43]; however, there is no information on its evolution. Hearing loss was reported in 11 studies [1, 12, 13, 23, 24, 27, 34–37, 43]: three at the initial onset [13, 24, 27], and one at the onset of follow-up [24]. Ocular involvement was present in 15 studies [11, 12, 23, 24, 26–30, 35−38, 40]. Uveitis and episcleritis were only mentioned once [35]. Laryngeal involvement was mentioned in four studies [11, 27, 31, 35]. Tracheobronchial involvement was found in five studies [11, 27, 28, 35, 36]. Laryngotracheitis was present in four articles [1, 24, 30, 37]. Costochondritis was found in nine articles [24, 27, 30, 33, 35, 37, 38, 41, 43], those being only two classified in initial onset [27] and follow-up onset [24]. Subglottic stenosis was found in three articles [1, 26, 36]. Neurological disorders were present in many articles, specifically in 13 studies [11–13, 23, 27–30, 34, 37–39, 42], of which two mentioned initial onset [23, 27] and only one mentioned follow-up onset [23]. Cardiac disorders were present in only two studies [23, 27], each of which described it as an initial onset [27] or follow-up onset [23]. Renal disorder was mentioned in eight articles [11, 12, 23, 24, 29, 37, 38, 42], but only one collected data on the follow-up onset [23]. Tracheal involvement, vestibular disorders, and bronchitis were not observed in any of the studies.

Auricular chondritis at initial onset was observed in 55.6% of patients [27], 74% [23, 24] developed as a follow-up symptom, and 67.3% presented with this symptom [1, 11, 23–25, 27, 28, 30, 31, 33–38, 40]. A median of 48.1% of patients presented septum nasal chondritis [11, 25, 26, 28–30, 33, 34, 36–40, 42, 43]. Ocular involvement affected 39.8% of patients [11, 12, 23, 24, 26–30, 35−38, 40]. Costochondritis occurred in 32.4% [24, 27, 30, 33, 35, 37, 38, 41, 43], 2.4% initially [27] and 15% eventually developed this symptom [24]. Neurological disorders were generally present in 12.8% [11–13, 28−30, 34, 37–39, 42], initially 3.5% [23, 27], and 9.6% of patients [23] at follow-up. Renal disorders generally occurred in 4.9% of cases [11, 12, 24, 29, 37, 38, 42]; none of the studies brought data from the initial onset, but follow-up was 9.2% [23]. Arthralgia occurred in 45% [11–13, 35] of patients, 7.1% had initial onset [13], and none were described as follow-up. Arthritis occurred in 37.7% [1, 24, 25, 27, 31, 35, 37, 43], 5.6% initial [24, 27], and 56% follow-up [24]. Nasal bridges account for 33.9% of the cases [1, 23]. Hearing loss was generally 41.7% [1, 12, 23, 24, 27, 34–37, 43], 5.8% at the initial follow-up [13, 24, 27], and 25% at the follow-up [24]. Uveitis generally occurs in 13% of patients [35], whereas episcleritis occurs in only 36% of patients [35]. Laryngeal involvement was observed in 33.3% [11, 27, 31, 35]. Tracheobronchial involvement affected 59.6% of patients [11, 27, 28, 35, 36]. Laryngotracheitis occurs in 68% of the cases [1, 24, 30, 37], whereas subglottic stenosis occurred in 11.4% [1, 26, 36]. Cardiac disorders occurred in 7.1% of the cases [23], initially 6.3% [27], and 7.1% at follow-up [23]. Bronchitis was not observed in any data (initial, general, and follow-up); the same was observed for vestibular disorders, septum nasal chondritis, ocular involvement, nasal bridge, uveitis, episcleritis, laryngeal involvement, tracheobronchial involvement, laryngotracheitis, or subglottic stenosis.

## Discussion

Among the 25 selected articles reviewed in this study, the clinical manifestations addressed were predominantly the classical symptoms of RP that were cumulatively presented. However, there has been limited discussion on other symptomatology and comorbidities that may accompany RP. Nonetheless, for full comprehension of RP progression, its clinical presentation must be thoroughly detailed, as in the present study. In this context, our current demographic study offers novel insights by providing a detailed temporal visualization of symptoms and treatment progression among patients with RP. This approach enriches our understanding of clinical evolution and treatment of this disease.

In this study, several significant strengths were identified. First, it involved a substantial sample size drawn from a continent with a limited number of clinical studies, thereby filling a critical gap in literature. Moreover, this study distinguishes itself from the existing literature by addressing a wide range of general aspects including clinical presentation, treatment, outcomes, comorbidities, and complications, thus contributing to a more holistic view of the disease under investigation.

In the literature review, data collection was conducted without applying the exclusion criteria based on the year of publication, which allowed the inclusion of studies spanning a wide temporal range. This approach provides a broader perspective by capturing the evolution of knowledge and practices over time. By including both older and more recent studies, this review offers a comparison of the scientific progress across different years. Additionally, it allowed the identification of research gaps, underexplored symptoms, and so on. As for most of the initial symptoms, the current study had almost twice the number of cases compared with the selected articles. This significant difference highlights the robustness of our initial data collection and the thoroughness with which we identify and document the early symptoms.

As for most of the initial symptoms, the current study had almost twice the number of cases compared to the selected articles. This significant difference highlights the robustness of our initial data collection and the thoroughness with which we identified and documented early clinical manifestations. However, regarding follow-up symptoms, our study included nearly half of the number of patients with general symptoms reported in the selected articles. This discrepancy could be attributed to several factors, including differences in the study design, population characteristics, and criteria used for symptom tracking. Additionally, we were unable to identify a consistent pattern in the general symptom category, unlike in other symptom categories where clear trends were observed. This lack of a pattern could suggest a higher variability in how general symptoms manifest and progress over time, or it might indicate that these symptoms are influenced by a broader range of external factors.

The sample composition in this study was predominantly white and female. In contrast, only nine of the 25 studies reviewed had the majority of female participants. Regarding ethnicity, none of the studies were from South America and most of them were from Asia. This lack of representation from Latin America highlights the significance of our study as it adds valuable insight into the understanding of the disease in the South American population. The absence of data from other demographic groups in the reviewed studies limited the generalizability of their findings, underscoring the importance of more diverse research in this field.

Regarding the demographic characteristics and onset of RP, the female-to-male ratio was 1.4:1, which was lower than the average ratio of 1.9:1 reported in the selected articles. Additionally, the age at diagnosis in our sample was 40 years, whereas the average age in the selected articles was 46.5 years.

The analysis of cardiovascular risk in patients with RP revealed significant differences between the selected patients and those in other studies in which comorbidities were inadequately addressed, hampering data analysis. However, systemic arterial hypertension was significantly more prevalent in our study (48.9%) than in the selected studies (9.6%). Dyslipidemia was also elevated in our sample (46.9%). The prevalence of DM was more than double in our patients (31.9%) compared with that in the data collected (13.4%). Regarding heart failure, our data showed a low prevalence (2.1%), whereas the prevalence of myocardial infarction and stroke was zero in our sample compared to 3.5 and 2.1 in other studies, respectively. Finally, the prevalence of smoking was lower in our patients (21.3%) than that in other studies (40.7%). These findings highlight the need for a personalized approach to manage cardiovascular diseases and their risk factors in patients with RP.

RP is characterized by episodic and unpredictable recurrence. Despite its recurrent nature, almost half of the patients achieved a controlled disease status at their last follow-up, with specific data showing 46.8% in remission, 29.8% with active disease, and 25.5% with controlled disease. This highlights the complexity of RP’s clinical trajectory of RP. The ability to attain disease control in a significant proportion of patients demonstrates advances in therapeutic approaches and potential for long-term management.

This study has some limitations. Firstly, the research design may have introduced a selection bias, as participants were recruited from a tertiary care setting. This population likely includes individuals with more severe symptoms or delayed diagnoses, potentially exaggerating symptom severity and skewing the representation of typical disease progression. Secondly, our samples were not subjected to *UBA1* (Ubiquitin Like Modifier Activating Enzyme 1) testing, which could have helped differentiate conditions such as VEXAS (Vacuoles, E1 enzyme, X-linked, Autoinflammatory, Somatic) syndrome. Lastly, we relied on physician judgment to determine disease status rather than using established metrics like the Relapsing Polychondritis Disease Activity Index (RPDAI).

## Conclusions

The reviewed articles described only classical manifestations of the disease but poorly described other clinical manifestations and comorbidities that can occur in RP. A demographic study in Brazil provided new insights by visualizing symptoms and treatment progression, and enhancing the understanding of the disease’s clinical evolution, outcomes, and comorbidities.

## Data Availability

No datasets were generated or analysed during the current study.

## Abbreviations

MP: Methylprednisolone
RP: Relapsing polychondritis
*UBA1*: Ubiquitin Like Modifier Activating Enzyme 1
VEXAS: Vacuoles, E1 enzyme, X-linked, Autoinflammatory, Somatic
RPDAI: Relapsing Polychondritis Disease Activity Index
